# Advances in locoregional therapy for hepatocellular carcinoma combined with immunotherapy and targeted therapy

**DOI:** 10.1016/j.jimed.2021.05.002

**Published:** 2021-05-15

**Authors:** Jian Xue, Hongbo Ni, Fan Wang, Ke Xu, Meng Niu

**Affiliations:** Department of Interventional Radiology, The First Affiliated Hospital of China Medical University, Shenyang, Liaoning, 110000, China

**Keywords:** Hepatocellular carcinoma, Locoregional therapy, Immunotherapy, Targeted therapy, Combination therapy

## Abstract

Locoregional therapies (LRTs) of hepatocellular carcinoma (HCC) represented by ablation and TACE has become the main means for the clinical treatment of unresectable HCC. Among these, TACE is used throughout the stage Ib to IIIb of HCC treatment. In recent years, immunotherapy led by immune checkpoint inhibitors has become a hot direction in clinical research. At the same time, targeted drugs such as Sorafenib and Apatinib have played an important role in the treatment and complementary therapy of advanced HCC, and their clinical application has been quite mature. HCC is the sixth most common malignant tumor in the world. When it comes to its treatment, different therapies have different indications, and their individual efficacies are not satisfactory, which makes the exploration of the use of combination therapy in HCC treatment become a new trend. In this paper, the status of the three therapies and the progress of their combined application are briefly reviewed.

## 1 Introduction

Primary hepatocellular carcinoma (HCC) is associated with a high mortality rate,^1^ and is the second leading cause of cancer-related deaths.^2^ Approximately 700,000 people worldwide die from hepatocellular carcinoma every year.^3^ Since early symptoms of HCC are not obvious, most patients are often diagnosed at the intermediate or advanced stages, and only 20%–30% of patients have surgical indications.^4^ Therefore, exploring effective treatments for advanced HCC is an important research area. Transcatheter arterial chemoembolization (TACE) is currently the most widely used and effective non-surgical treatment for liver cancer, although local ablations have also been used in clinical practice in recent years. However, locoregional therapy does not often result in ideal therapeutic outcomes, due to the aggravation of local tumor hypoxia after treatment and the resulting tumor progression or recurrence. In recent years, immunotherapy has played an increasingly important role in the systemic treatment of liver cancer, and progress has been made in basic and clinical research of liver cancer immunotherapy. Nevertheless, the objective response rate of immunotherapeutics, represented by PD-1/PD-L1 and CTLA-4 antibodies, is low.^5^ Targeted therapeutic drugs represented by tyrosine kinase inhibitors (TKIs), such as sorafenib, have been approved by the FDA for clinical use.^6^ However, different treatments have different indications and efficacies in patients at different stages of HCC. These therapeutic bottlenecks suggest that a combination of these therapies may lead to more effective tumor responses, longer survival, and longer time to tumor progression. This paper reviews recent studies and reports on the progress in the development and comprehensive use of these therapies. The findings are summarized below.

## 2 Locoregional therapies (LRTs): the “captain” of treatment of unresectable liver cancer

Image-guided locoregional therapy (LRT) is an important component of therapies for hepatocellular carcinoma (HCC),^7^ particularly in advanced unresectable liver cancer. LRTs are divided into percutaneous ablation and vascular interventional therapy. The latter consists of Transcatheter Arterial Infusion chemotherapy, Transcatheter Arterial Embolization, Transcatheter arterial chemoembolization (TACE), and Transarterial Radioembolization.^8^

### 2.1 Percutaneous ablation

Liver cancer ablation methods vary, but generally include physical and chemical ablations. Physical ablations include radiofrequency ablation (RFA), microwave ablation (MWA), high-intensity focused ultrasound (HIFU), laser ablation (LA), cryoablation, and irreversible electroporation (IRE). Chemical ablations include percutaneous ethanol injection (PEI). Cryoablation, IRE, and PEI are nonthermal ablation methods, while the remaining methods cause coagulative necrosis of tumor tissue using heat and are collectively referred to as thermal ablations. RFA and MWA are widely used in local ablation therapy, and their effects are definite. When the lesion is ​> ​2 ​cm in size, RFA has a better effect than PEI. MWA may be more effective for adjacent large blood vessels or large tumors. However, there is currently no significant difference between RFA and MWA in terms of local efficacy or complications.^8^ RFA, MWA, and cryoablation are described in subsequent sections of this review.

#### 2.1.1 Radiofrequency ablation (RFA)

Between 1990 and 1995, Rossi and McGahan et al. conducted animal experiments and clinical studies on radiofrequency ablation of liver cancer, which was first reported as a clinical treatment in 1995. RFA, surgical resection, and liver transplantation are the most commonly used treatments for early stage liver cancer, and RFA is currently recognized as the main ablation method for HCC tumors less than 5 ​cm.^9^ Compared with surgery, it has fewer complications and lower costs, but clinical studies have not shown clear differences in survival,^10^^,^^11^ with some of the studies showing contradicting results.^7^

#### 2.1.2 Microwave ablation (MWA)

MWA can increase the temperature of local tissue using microwaves, leading to coagulative necrosis and thus treat liver cancer. Potretzke et al.^12^ showed that microwave ablation has significant advantages over RFA because it reduces the progression of local tumors. Recent immunological studies^13^ show that MWA has significant immune-related effects on patients with liver cancer and can enhance specific tumor immune responses.

#### 2.1.3 Cryoablation

Cooper^14^ proposed the idea of cryotherapy for liver cancer treatment as early as 1963, and this was first achieved through close cooperation between radiologists and surgeons. Currently, there are two commonly used cryoablation methods: argon-helium knife and liquid nitrogen. The principle is based on reducing the local temperature of the tumor and inducing tissue freezing and blood vessel damage using the Thomson effect.^15^ Cryoablation has several advantages compared with RFA and other hyperthermia-based methods, including the ability to produce a larger and more accurate ablation area.^16^ Kim et al.^17^ showed that cryoablation had a high technical success rate in the treatment of perivascular hepatocellular carcinoma. Only 6.9% of the patients had peripheral vascular thrombosis, and there was no infarction or other major vascular complications. The cumulative local tumor progression (LTP) rate at two years was 14.6%.

### 2.2 Vascular interventional therapy

At present, the main interventional therapy for hepatocellular carcinoma is vascular interventional therapy, with transcatheter hepatic arterial chemoembolization (TACE) being the most widely used method. TACE is the first choice for patients with liver cancer who cannot be treated with radical therapy. TACE includes conventional TACE (cTACE) and current running TACE of drug-eluting beads (DEB-TACE).

#### 2.2.1 Conventional TACE (cTACE)

Contrary to the 75% portal vein and 25% hepatic artery composition of the blood supply to normal liver tissue, 85%–90% of the blood supply in liver cancer lesions comes from the hepatic artery, while the remaining 10%–15% comes from the portal vein. This indicates that TACE can effectively kill tumor tissue while minimizing damage to the normal liver. TACE is the recommended treatment for stage B Barcelona Clinic Liver Cancer (BCLC). It can significantly increase the concentration of drugs in tumor tissues and block blood supply to the tumors. Chemotherapy and embolization work together to produce the best results. Furthermore, TACE can reduce liver cancer lesions that cannot be transplanted to the Milan standard. Clinically, a suspension of chemotherapeutic drugs and iodized oil is generally used in TACE. After injection into the hepatic artery, it is deposited in the tumor tissue where it can remain for more than one year because of the siphon effect of tumor tissue on iodized oil. Although the median survival time (MST) of patients treated with TACE is more than 2.5 years,^18^^,^^19^ the repeated use of chemotherapeutic drugs increases toxicity. Moreover, arterial embolization exacerbates the ischemia and hypoxia of the tumor, contributing to the tumor microenvironment (TME). This allows the tumor not only to evade immune surveillance, but also to be further enhanced by glycolysis, leading to faster tumor growth and, eventually, recurrence and metastasis.

#### 2.2.2 TACE of drug-eluting beads (DEB-TACE)

A new chemoembolization method, drug-eluting beads (DEB), has been recently introduced and is gradually being applied in clinical treatment. Both iodized oil and DEB act as carriers of chemotherapeutic drugs such as adriamycin and DDP, thereby allowing a slow-release effect that produces sustained chemical toxicity in tumor cells. While DEB-TACE is considered a more standardized and reusable method for drug release,^20^ studies have shown that bile duct injury, bile tumor, and adverse events associated with liver function injury are significantly higher following DEB-TACE than cTACE, and are more obvious in patients with severe cirrhosis.^21^ However, there were no statistically significant differences in anti-tumor effect between the two methods.^22^, ^23^, ^24^ A meta-analysis by Zou et al.^25^ in 2016 showed that DEB-TACE had a higher rate of complete remission and overall survival (OS) in HCC patients than cTACE; DEB-TACE was also safer and had fewer adverse events than cTACE. However, the study recommended that the results should be interpreted with caution as the comparison of efficacy and side effects between DEB-TAC and cTACE is controversial.

#### 2.2.3 Transarterial radioembolization

Transarterial radioembolization is an *in vivo* radiotherapy technique in which radiotherapy and embolization are performed simultaneously. It is clinically called selective internal radiotherapy (SIRT) and forms a part of local radiotherapy. It shares the same principle with TACE, except that chemotherapeutic drugs are replaced with either radionuclide yttrium 90 (Y90) or Lutetium 177 (Lu177), with the former being more common in practical applications. By placing radioisotope-loaded microspheres into the liver via the hepatic artery and emitting β-rays at an average distance of 0.25 ​cm, this radiotherapy directly kills tumor cells at close range. It has fewer side effects than traditional *in vitro* radiotherapy and is especially suitable for patients with large tumors, inoperable or poor TACE, or ablation effects. A systematic review^26^ of 14 clinical studies and 3 meeting abstractions involving 722 patients showed that the median survival was 9.7 months for all patients receiving Y90 transarterial radioembolization. The median OS was 12.1 months and 6.1 months in Child-Pugh class A and class B patients, respectively, while the median OS was 6.1 months and 13.4 months in patients with main and branch portal vein tumor thrombosis(PVTT), respectively, indicating that radioembolization is a safe and effective method for treating HCC and portal venous thrombosis (PVT). The use of Y90 was also shown to prolong survival time.^27^ In this study, 34 of 185 patients (18%) with locally advanced PVT hepatocellular carcinoma who received Y90 radioembolization had a survival time ≥18 months. Chow et al.^28^ compared the efficacy of Y90 radioembolization with sorafenib in patients with locally advanced liver cancer in the Asia-Pacific region, and found no significant differences in OS between patients receiving the two treatments. A multicenter phase 3 trial (NCT01482442) in France^29^ also found no significant differences between patients in the two treatment arms.

### 2.3 Combined use of LRTs

Due to the limited effects observed with single treatments, researchers have recently begun to explore combinations of two or more local treatments; mainly the combination of TACE and various local ablation procedures, and their outcomes have been widely reported. A meta-analysis by Wang et al.^30^ showed that the 6-month, 1-year, 2-year, and 3-year survival rates for patients in the TACE+PEI group were significantly better than for patients in the TACE group. In terms of the AFP decline rate and the tumor volume reduction rate (>50%), the TACE+PEI group was superior to the TACE group, and the incidence of adverse reactions was lower in this group than in the TACE group. A similar study by Fu et al.^31^ also confirmed that combining TACE and PEI in the treatment of unresectable liver cancer could improve survival rate, reduce local tumor recurrence, and reduce AFP level and tumor size.

Theoretically, TACE and RFA have complementary roles: TACE can block tumor blood vessels and reduce the “heat sink effect” during ablation, while lipiodol deposition can trace the HCC lesion, providing a clear target for ablation. In addition, the thermal effect of ablation can improve the sensitivity of liver cancer to chemotherapy drugs and enhance the efficacy of TACE. However, Kim et al.^32^ compared the safety and efficacy of combining TACE and RFA versus using TACE or RFA therapies individually for treatment of small hepatocellular carcinoma (SHCC), and found that hospitalization duration for patients in the TACE+RFA group was longer than for patients in the TACE only or RFA only groups. The incidence of discomfort was significantly lower in patients in the TACE+RFA group, and the incidence of postoperative complications was also lower in the TACE+RFA group. There were no statistically significant differences in the incidence of major complications in the three groups. There were no statistically significant differences in Child-Pugh scores in the three groups (P ​= ​0.162) at the 1-month follow-up. The tumor response in patients in the TACE+RFA group at 1 month, 6 months, and 1 year was similar to that in patients in the RFA group, but was superior to that in patients in the TACE group. Thus, tumor efficacy of TACE combined with RFA is similar to that achieved with RFA monotherapy but is superior to that achieved with TACE monotherapy. Therefore, TACE+RFA treatment may be required for specific patients with SHCC, especially those who do not meet the RFA monotherapy conditions. However, combining TACE and RFA was shown to be safe and effective in the treatment of medium and large hepatocellular carcinomas and had the long-term effect of delaying tumor progression and improving progression-free survival (PFS) and OS.^33^

Yuan et al.^34^ conducted a comparative study to determine whether there was a difference in efficacy between TACE-RFA and TACE-MWA treatments, and found no significant difference in the short-term efficacy in the treatment of medium and large primary HCC between patients receiving TACE-RFA and those receiving TACE-MWA. However, when the tumor diameter was >5 ​cm, patients in the TACE-MWA group had better efficacy than those in the TACE-RFA group. Although there was no significant difference in the one-year cumulative survival rate and the tumor-free survival rate between patients in the two groups, postoperative liver function damage was significantly lower in patients in the TACE-RFA group than in those in the TACE-MWA group.

In addition, a 2016 meta-analysis^35^ of TACE and various ablation procedures compared the effects of single versus combination therapy in 11 randomized controlled trials, and found that the one-year mortality rate was higher with single interventional therapy than with combination therapy. There were no differences in the one-year mortality rates between patients receiving TACE and TACE+PEI. However, 3-year mortality was significantly reduced, while one-year mortality was higher following treatment with RFA alone than with TACE+RFA. In summary, TACE combined with percutaneous ablation can, to some extent, improve the survival rate of patients with unresectable HCC.

Local ablations can also be used in combination. Azab et al.^36^ analyzed the treatment of 90 cases of hepatocellular carcinoma divided into RFA, PEI, and combined treatment groups. The results showed that after the first treatment, the rate of complete ablation in the combined treatment group (87.9%) was significantly higher than that in the RFA group (54.54%). After the second treatment, the success rate of complete ablation in the combined treatment group was 97.0%, while the success rate of complete ablation in the RFA group was 84.8%. In the PEI group, 75% of patients experienced complete ablation, and the 1.5-year survival rate in the combined group was also better than that in the other two groups.

## 3 Immunotherapy: the “adjutant” of LRTs

HCC can induce tumor immune tolerance through various mechanisms, evade immune damage, and eventually develop and metastasize.^5^ Therefore, in recent years, various immunotherapy strategies, from molecular, cellular, metabolic, and TME, have been developed. Immunotherapy is a new development in the treatment of HCC that is likely to play a major role in the future, both in monotherapy and combination therapy. Conventional immunotherapy includes immune checkpoint inhibitors, adoptive cell therapy (ACT), oncolytic virus therapy, cytokine therapy, and tumor vaccine therapy. The first two therapies in this list are widely used in the treatment of HCC and have been researched extensively; the newly discovered metabolic checkpoint inhibitors are introduced in subsequent sections.

### 3.1 Immune checkpoint inhibitors

Immune checkpoints are a series of regulatory molecules expressed on immune cells that can regulate the degree of immune activation and generally act as inhibitors. The immune checkpoint plays an important role in maintaining self-tolerance and preventing autoimmune diseases. Liver cancer cells express some substances that activate immune checkpoints, so that tumor antigens cannot be presented to T cells, while at the same time inhibiting T cell functions, ultimately leading to immune escape of the tumor. Immune checkpoints include programmed death receptor-1 and its ligand (PD-1-PD-L1/PD-L2), cytotoxic T lymphocyte-associated protein-4 (CTLA-4), mucin molecule-3 (Tim-3), lymphocyte activation gene-3 (LAG-3), and B and T lymphocyte attenuator (BTLA).^37^ PD-1/PD-L1 and CTLA-4 are the most widely used in HCC clinical immunotherapies.

Treatment involving the PD-1/PD-L1 pathway has developed rapidly in recent years. Currently, anti-PD-1/PD-L1 antibodies are used for systematic treatment, which is based on the principle that PD-1 monoclonal antibodies activate functionally depleted T cells by blocking the immunosuppressive response mediated by the PD-1 pathway, recovering the tumor-killing effect of T cells.^38^ Nivolumab, pembrolizumab, atezolizumab, and camrelizumab were approved by the U.S. Food and Drug Administration (FDA) as second-line treatments for liver cancer in 2015.^39^

Cui et al.^40^ carried out a retrospective analysis of 55 patients with advanced primary HCC who received anti-PD-1 drug treatment, and found an OS of 15 months and a median PFS of 10 months. No patient had complete response (CR) and only 12 patients (22%) had partial response (PR). The overall response rate (ORR) was 22% and the disease control rate (DCR) was 89%. The total incidence of adverse reactions was 61.8%, but most were relieved after treatment. A retrospective study from three German centers^41^ explored the feasibility and safety of nivolumab for the treatment of advanced hepatocellular carcinoma. Of the 34 patients assessed, 20 (58.8%) died, 2 (5.9%) had grade 3 adverse reactions, and the total effective rate was partial remission in 4 cases (11.8%) and stable in 8 cases (23.5%). The median OS of the entire cohort was 7.5 weeks. This indicates that further evaluation in patients with advanced liver disease is needed, as the efficacy of nivolumab may be limited. As the first approved drug, nivolumab is currently in phase 3 clinical trials for HCC treatment. Nordness et al.^42^ reported a case involving treatment with nivolumab before hepatocellular carcinoma transplantation, which resulted in fatal acute liver necrosis immediately after the operation due to the profound immune response that nivolumab caused.

Studies on pembrolizumab in patients with advanced HCC have also been conducted. Finn et al.^43^^,^^44^ conducted a multi-phase clinical trial of this drug (KEYNOTE-224, KEYNOTE-240). The non-randomized, multicenter, open-label, phase 2 KEYNOTE-224 trial showed an objective response in 18 of 104 patients (17%). The best overall responses were: 1 (1%) complete response and 17 (16%) partial responses; meanwhile, 46 (44%) patients had stable disease and 34 (33%) had progressive disease. The randomized, double-blind, phase 3 study, KEYNOTE-240, evaluated the efficacy and safety of pembrolizumab. It found that median OS was 13.9 months for pembrolizumab and 10.6 months for placebo, while median PFS was 3.0 months for pembrolizumab and 2.8 months for placebo at the first interim analysis and 3.0 months versus 2.8 months at final analysis.

Recently, a multicenter randomized phase 2 trial found that camrelizumab had anti-tumor activity and controlled toxicity in the pre-treatment of advanced hepatocellular carcinoma patients in China.^45^ In addition, Qin et al.^46^ compared the efficacy, safety, and tolerability of tislelizumab, another PD1 monoclonal antibody, with sorafenib as a first-line treatment for unresectable liver cancer.

Tremelimumab is an anti CTLA-4 all-human monoclonal antibody. Significant activation of T cell responses were observed in patients with hepatocellular carcinoma who were treated with tremelimumab.^47^ However, Sangro et al.^48^ concluded in a separate study that the safety, and antitumor and antiviral activities of tremelimumab in patients with advanced HCC who had post-hepatitis C cirrhosis needed to be studied further.

Ipilimumab is another monoclonal antibody that can effectively block CTLA-4 molecules. Check Mate 040 multicenter randomized clinical trials (NCT01658878)^49^^,^^50^ showed that its combination with nivolumab in patients with previous sorafenib treatment had controllable safety, promising objective response rates (up to 27%) and persistent response.

### 3.2 Adoptive cell transfer (ACT) therapy

Adoptive cell transfer (ACT) therapy refers to the extraction of immune cells from the tumor or peripheral blood of the patient for *in vitro* culture and subsequent re-injection into the patient's body. It is used mainly in the treatment of melanoma, hematologic tumors, lymphoma, and other non-solid tumors, and is currently the most effective method for treating patients with metastatic melanoma.^51^ Its application in solid tumors also has good prospects. ACTs include tumor infiltrating lymphocytes (TILs), dendritic cells (DCs), natural killer (NK) cells, cytokine-induced killer (CIK) cells, T Cell Receptor T Cells (TCR-T Cell), and Chimeric Antigen Receptor T Cell (CAR-T Cell). Dendritic cells are the main specialized tumor antigen-presenting cells *in vivo*, and can induce antitumor immune responses of specific cytotoxic T lymphocytes (CTL) by impinging sensitized DCs with tumor-associated antigens or antigen peptides *in vitro* and then transfusing them back into the tumor patients. IL-12 sensitized DC immunotherapy is a promising method for liver cancer treatment.^52^ In a randomized controlled trial of CIK cells with TACE and RFA, CIK cell therapy reduced recurrence and metastasis in HCC patients, but there was no improvement in OS.^53^ A retrospective study^54^ evaluated the prognosis and factors influencing DC-CIK cell therapy following the use of TACE for HCC. The OS time for HBV-infected HCC patients in the study group (TACE+DC-CIK cell therapy) was significantly longer than for patients in the control group (TACE only). The PD-L1 expression level in tumor tissues was significantly negatively correlated with relapse-free survival (RFS) and OS. T cells in the tumor microenvironment are inhibited by various mechanisms, particularly, lack of synergistic stimulation molecules and MHC molecules, making it difficult to recognize and kill tumor cells. TCR-T and CAR-T cells were therefore developed. Unlike hematologic malignancies, solid tumors are less sensitive to CAR-T cell therapy, partly due to the reduced aggregation of therapeutic T cells at tumor sites.^55^ In recent years, studies have been conducted on the application of CAR-T cell surface target modification in the treatment of liver cancer, with promising results.^56^, ^57^, ^58^

### 3.3 Metabolic checkpoint inhibitors: recent advances in immunotherapy

Metabolic differences which might play a guiding role in tumor immunotherapy were identified between tumor cells and T cells. In recent years, studies on the three major metabolic pathways of various cells in the tumor immune microenvironment have gained increased attention, with metabolic checkpoint studies being most popular. Metabolic checkpoints refer to some important enzymes or receptors in metabolic pathways, or even some metabolic intermediates, whose activity levels directly affect the immune function of immune cells and can regulate the anti-tumor activity of T cells. Currently, glutamine (Gln) inhibitors, Indoleamine-2,3-dioxidase (IDO) inhibitors, and acetyl-CoA cholesterol acetyltransferase (ACAT) inhibitors are of most interest. These inhibitors remove T cell inhibition through metabolic pathways, improve their activity, and kill tumor cells.

Leone et al.^59^ performed a step-by-step analysis and validated a glutamine multi-target inhibitor called JHU083 based on 6-diazo-5-oxonorleucine (DON) in mouse models of colon cancer, lymphoma, colon cancer, and melanoma. They found that JHU083 could disrupt the low-oxygen, acidic, and nutrition-deficient tumor microenvironment formed by the Warburg effect, through metabolic pathways, thus inhibiting the growth of tumor cells, significantly upregulating the oxidative metabolism, life, and activity of CD8^+^ T cells, restoring tumor immunity, and achieving anti-tumor activity. Although no animal experiments have been conducted to study the treatment of hepatocellular carcinoma, the application of JHU083 and anti-PD-1 antibody in hepatocellular carcinoma should be researched, as the combination of JHU083 and anti-PD-1 antibody can improve the survival rate of mice and reduce tumor volume.

In addition, IDO also plays an important role in mediating the immune tolerance of HCC through a variety of mechanisms. Asghar^60^ showed that IDO is associated with invasiveness of liver cancer and poor prognosis in patients, thus IDO can also be used as a potential therapeutic molecule.

## 4 Targeted therapies: “missile weapons” killing the tumor

The clinical application of targeted therapy for liver cancer is well developed, and mainly includes multi-target tyrosine kinase inhibitors (TKIs), represented by sorafenib, and vascular endothelial growth factor inhibitors (VEGFIs), represented by apatinib.

TKIs include sorafenib, which is recognized as the first-line treatment for HCC, and lenvatinib, regorafenib, and ramucirumab. These drugs have been approved by the FDA in recent years for use in the clinical treatment of HCC.^39^ Regorafenib has been used in patients for whom sorafenib treatment failed^61^, ^62^, ^63^, ^64^ and was approved as a second-line treatment for hepatocellular carcinoma in China in 2018.

Ramucirumab specifically blocks blood vessel growth factor receptor II (VEGFR-II), which is its only target; thus, it can also be incorporated into the anti-angiogenesis drugs (VEGFI). A global randomized phase 3 study, REACH-2 (NCT02435433), found that in patients with advanced hepatocellular carcinoma and AFP ​≥ ​400 ​ng/mL, treatment with the second-line drug ramucirumab significantly increased OS compared with placebo. The drug was also shown to be effective in a Japanese subpopulation.^65^ However, in their review, Roviello et al.^66^ explored whether using remolumab as a second-line drug the treatment of advanced liver cancer would lower its potential for use in individualized treatment.

**Fig. 1:**
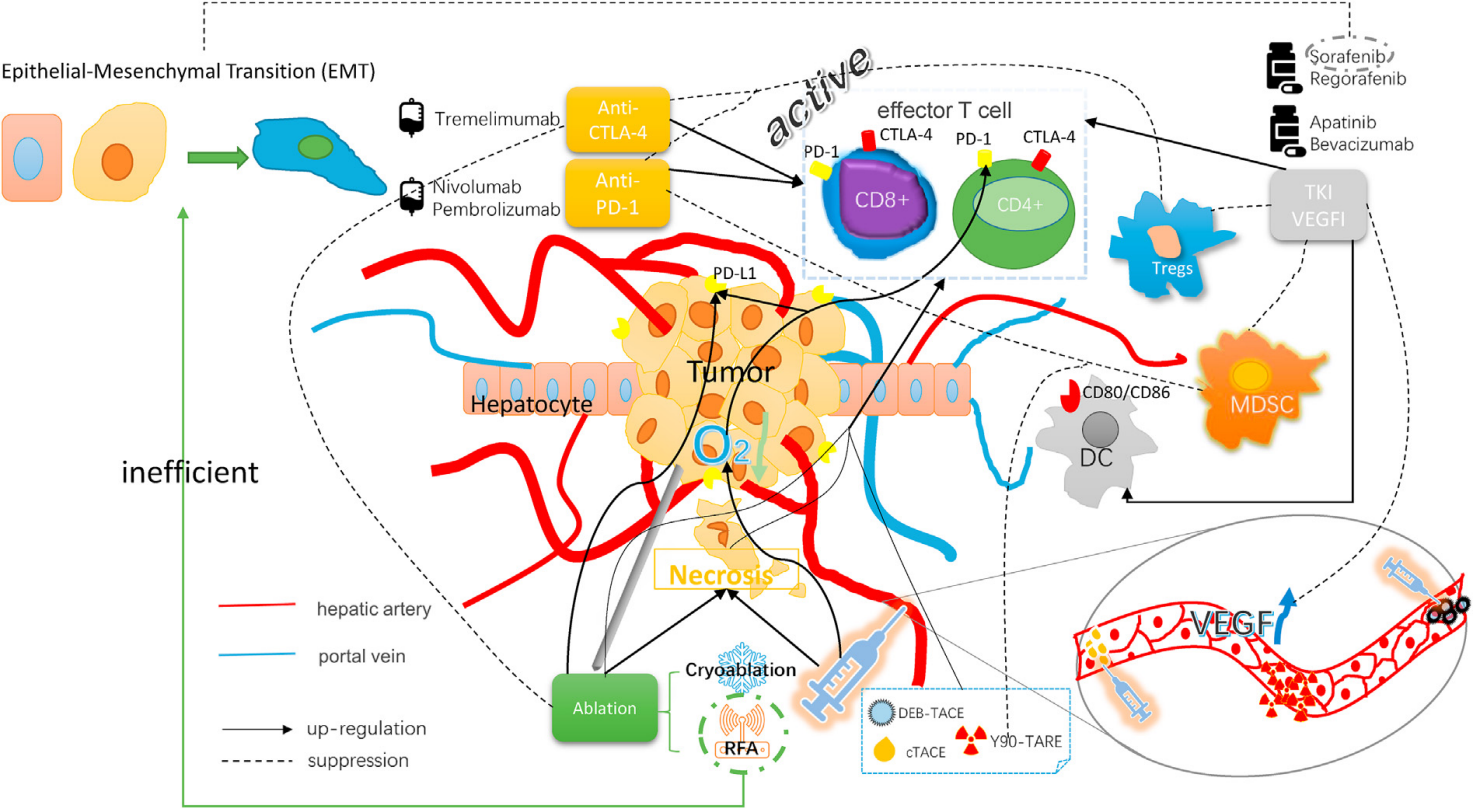
LRT, immunotherapy and targeted therapy can produce synergistic effects in a variety of ways. TACE and ablation lead to tumor necrosis, enabling effector T cells to activate, recognize, and kill tumors. RFA can induce epithelial mesenchymal transformation (EMT) of tumor cells, and sorafenib can inhibit this effect. Local VEGF levels are elevated following TACE, thus anti-VEGF drugs may play a counter role. Following TACE, tumor hypoxia and PD1/PDL1 expression are increased, which provides an opportunity for anti-PD1 therapy; RFA has similar effects. In addition, the combination of LRTs and anti-PD1 therapy can reduce the aggregation of myeloid-derived suppressor cell (MDSCs) and the proportion of Tregs. Targeted drugs can also promote the activation and aggregation of DCs, while anti-CTLA-4 therapy can remove the inhibition of the antigen presentation process, enabling T cells to fully play their role. Immune checkpoint inhibitors can activate functionally depleted T cells and restore the tumoricidal effect of T cells by blocking the corresponding pathway-mediated immunosuppressive response. Anti-VEGF drugs can cause blood vessel normalization, inhibit VEGF signaling pathways, restore DC function, enhance the infiltration and function of immune cells, and reduce the infiltration of Tregs.

VEGFI can normalize blood vessels and plays several functions by inhibiting the VEGF signaling pathway, including restoring the function of antigen presenting cells (DCs cells), enhancing the infiltration and function of effector immune cells, and reducing the infiltration of immunosuppressive cells such as Tregs^67^ (Fig. 1). In addition to the above multi-target TKIs, which also have a VEGFI effect, the main clinical antiangiogenic drugs include apatinib and bevacizumab. A phase 2 clinical trial (NCT03046979)^68^ examined the efficacy of apatinib as a first-line treatment for advanced HCC. The median OS and PFS were 13.8 months and 8.7 months, respectively. The most common treatment-related adverse events were proteinuria (39.1%), hypertension (34.8%), and hand-foot skin reactions (34.8%). In addition, He et al.^69^ showed that apatinib could achieve PFS and OS rates comparable to those achieved with sorafenib in patients with advanced HCC and even had a better objective response rate (ORR). Wattenberg et al.^70^ initiated bevacizumab treatment (5–10 ​mg/kg every 2–3 weeks) from 2008 to 2017 in 12 patients with advanced HCC who were intolerant or were at an advanced disease stage during sorafenib treatment. The drugs were well tolerated, with a median OS of 20.2 months, a median radiological time to progression (TTP) of 10.4 months, and a disease control rate of 54%. Thus, the application of bevacizumab in patients with advanced HCC warrants further clinical studies.

## 5 Immunotherapy associated with targeted therapy: to improve the efficacy

The dual blocking actions of PD-1 monoclonal antibody and VEGFI can enhance anti-tumor effects through a variety of mechanisms, showing a promising prospect for application in killing tumor cells.

On May 29, 2020, the FDA approved atezolizumab in combination with bevacizumab for the treatment of unresectable, locally advanced, or metastatic HCC that has not been systematically treated in advance.^6^ The results of a comparative study between atezolizumab-bevacizumab and sorafenib showed that the median OS for patients in the atezolizumab-bevacizumab group could not be estimated (the end point was not observed), but was 13.2 months for patients in the sorafenib group. The median independently assessed PFSs for the atezolizumab-bevacizumab combination and sorafenib were estimated to be 6.8 months and 4.3 months, respectively. There were differences in adverse reactions, with the incidence of bleeding being higher in atezolizumab-bevacizumab treated patients (25%) than in sorafenib-treated patients (17%). In addition, Lee et al.^71^ carried out a phase 1B study which showed longer PFS when atezolizumab was used in combination with bevacizumab than when atezolizumab was used alone. These results were confirmed by Finn et al.^72^ Therefore, combination therapy could be a promising treatment option for patients with unresectable HCC. On October 28, 2020, the Chinese National Medical Products Administration also approved this combination regimen for the treatment of patients with unresectable HCC who had not received prior systematic treatment.

Apart from this well-used combination, a new combination of camrelizumab and apatinib is also under study. Xu et al.^73^ showed that camrelizumab combined with apatinib showed good efficacy and controllable safety in both first and second line treatments of advanced HCC. The use of a combination of lenvatinib and pembrolizumab in patients with unresectable HCC has also been reported. In a phase 1 ​B open multicenter study, Finn et al.^74^ found that the lenvatinib-pembrolizumab combination regimen was well tolerated, and no unexpected safety issues were observed. Additionally, its toxicity could also be controlled by dose adjustment, interruption, and supportive therapy.

## 6 Trends in treating HCC: LRTs combined with immunotherapy or targeted therapy

TACE and ablation can induce liver tumor cell necrosis and activate T-cell responses.^75^, ^76^, ^77^ They can also increase PD-1/PD-L1 expression in the tumor microenvironment.^78^^,^^79^ Thus, anti-PD-1 drugs are therapeutic molecules. The combination of LRTs and anti-PD1 therapy can also reduce the aggregation of myeloid-derived suppressor cells (MDSCs) and the proportion of Tregs.^79^^,^^80^ (Fig. 1).

### 6.1 Vascular interventional therapies

Using TACE and radioembolization, multiple immune agents and targeted drugs can be combined to more effectively kill advanced unresectable HCC lesions. Two retrospective studies by Chapiro et al. and Jin et al. respectively^81^^,^^82^ reported that sorafenib combined with TACE was more effective at treating advanced liver cancer than sorafenib alone, while the results of a randomized controlled trial^83^ showed that the combination of sorafenib and TACE did not improve treatment efficacy in European, American, or Asian populations. Kudo et al.^84^ conducted a randomized, multicenter, prospective trial which showed that the median PFS in the group of patients treated with TACE and sorafenib was significantly longer than that in the group treated with TACE alone (25.2 months vs. 13.5 months, respectively; P ​= ​0.006). Median time to untreatable (unTACEable) progression (TTUP) without TACE treatment was also significantly prolonged in the sorafenib group (26.7 months vs 20.6 months, p ​= ​0.02). The one-year and two-year overall survival rates of TACE combined with sorafenib and TACE alone were 96.2% and 82.7%, and 77.2% and 64.6%, respectively. No unexpected toxic reactions were observed in the study. In addition, Li et al.^85^ and Wei et al.^86^ conducted a systematic review and meta-analysis of studies on the combined use of TACE and sorafenib for the treatment of unresectable hepatocellular carcinoma and found that combined therapy could prolong TTP and DCR in patients with unresectable HCC.

Anti-angiogenesis drugs can also be used in combination with TACE to improve their curative effects. Liu et al.^87^ analyzed the efficacy and safety of TACE combined with low-dose apatinib for the treatment of unresectable HCC in elderly patients. Their results showed that the median survival for patients in the experimental (combination therapy) group (26.0 months) was significantly longer than for patients in the control (TACE alone) group (20.0 months). The adverse reactions were higher in the experimental group than in the control group and were likely associated with taking apatinib. However, these reactions were generally reduced after symptomatic treatment. Shen et al.^88^ found similar results in their study which looked at the treatment of HCC with macrovascular invasion.

Although bevacizumab and apatinib are both anti-vascular drugs, one study^89^ showed no improvement in radiological tumor response and OS in hepatocellular carcinoma patients treated with bevacizumab and cTACE, with severe or even fatal sepsis and vascular side effects. Therefore, bevacizumab cannot be used as an adjuvant treatment for cTACE. However, the drug-loading convenience of DEB-TACE has recently been demonstrated. In addition to chemotherapy drugs, it can be used for loading new drugs such as SW43-DOX. It is therefore important to explore new delivery routes for immune-targeted drugs. For example, it should be considered whether a different outcome could be achieved if bevacizumab is combined with TACE via DEB load. Sakr et al.^90^ investigated the feasibility of loading bevacizumab in DEB as a way to reduce drug exposure in the systemic circulation, reduce drug toxicity and side effects, and improve efficacy. The results showed good biological activity after *in vitro* release; however, *in vivo* responses need to be explored further. Studies on the application of drug-eluting beads in drug load elution have also led to the development of new TKIs, such as sunitinib and Vandetanib.^91^^,^^92^

TACE can also be combined with immunotherapy. In addition to the above-mentioned cell therapy as the adjuvant therapy of TACE, a study by Nakamoto^93^ showed that an active immunotherapy strategy based on dendritic cells combined with TAE had good anti HCC tumor effect. The combination of checkpoint inhibitors and TACE is still under study. It is based on the principle that combining LRTs and immune checkpoint inhibitors can enhance the anti-tumor immune response.^94^ Several studies have examined combinations of different drugs and TACE, including nivolumab-TACE (NCT03572582),^95^ nivolumab-DEB-TACE (NCT03143270),^96^ and pembrolizumab-TACE (NCT03397654).^97^

As mentioned above, there was no significant difference between using Y90 radioembolization and sorafenib individually; therefore, the combination is expected to have different results. A study^98^ showed that OS and PFS achieved with this combination therapy are superior to those achieved by sorafenib alone. Lorenzin et al.^99^ found no evidence of liver cancer recurrence during 12 months of follow-up following treatment with a combination of the two therapies. However, Teyateeti et al.^100^ reported that the median OS and PFS were 12.4 months and 5.1 months, respectively, in patients with combined therapy, and 21.6 months and 9.1 months, respectively, in patients with radioembolization alone, and their OS might have been associated with serum AFP. Therefore, further studies on the effect of the combination therapies are needed. In addition, the combined application of transarterial radioembolization and PD1 monoclonal antibody is also currently under analysis: nivolumab (NCT03033446,^101^ NCT03380130^102^) and pembrolizumab (NCT03099564^103^).

### 6.2 Combinations with ablations

HCC progression and local recurrence rates were very high following RFA therapy alone, especially for larger tumors (d ​> ​3 ​cm).^104^ Therefore, a combination of local ablation and targeted therapy was considered as an alternative treatment. In 2015, de Stefano et al.^105^ confirmed the safety and efficacy of sequential RFA and sorafenib therapy in patients with HCC. The mechanism of action may involve sorafenib inhibiting the epithelial-mesenchymal transformation (EMT) of liver cancer cells after RFA deficiency and preventing the progression of liver cancer following RFA.^106^ However, Tang et al.^107^ used a mouse model to demonstrate that sorafenib can improve the efficiency of RFA, possibly through inhibition of angiogenesis. Thus, this combination therapy may have a potential effect. Kan et al.^108^ studied the use of sorafenib combined with percutaneous RFA for the treatment of medium-scale liver cancer. During the follow-up period, the recurrence rate was 56.7% in the combined treatment group and 87.5% in the radiofrequency-only group, and the median TTP was 17.0 months in the combined treatment group and 6.1 months in the RFA-only group, with most of the adverse events (AEs) in the combined treatment group being mild to moderate.

Ablation and immunotherapy are also a good combination. da Costa et al.^9^ reviewed the research progress on treatment with RFA combined with immunotherapy and found that compared with RFA alone, the combination therapy could improve the reactivity of anti-tumor T cells, significantly reduce the risk of recurrence, and improve survival rate. In addition, a cohort study (NCT01853618) demonstrated that tremelimumab could be safely and effectively used in combination with ablation for the treatment of advanced HCC.^109^ The principle is similar to TACE combined immunotherapy, in that ablation to kill the tumor activates the immune system, and immune checkpoint inhibitors enhance this effect. The study found a significant increase in the number of CD8+T cells in patients, with clinical benefit; the probability of progression-free survival at 6 months and 12 months in patients with refractory HCC was 57.1% and 33.1%, respectively, and the median time to tumor progression was 7.4 months. The median OS was 12.3 months. The combination of LRTs and immune checkpoint inhibitors is a potential new therapy for advanced HCC, and a study (NCT01853618) testing the treatment of BCLC stage B or C with tremelimumab combined with TACE, RFA, and cryoablation is currently in progress, and new results are expected to emerge.

### 6.3 Combination of multiple therapies for individualized treatment

Reports on the combined use of more than two therapeutic methods in locoregional therapies, immunotherapies, and targeted therapies have emerged in recent years. Peng et al.^110^ treated patients with advanced recurrent HCC with sorafenib alone or with sorafenib in combination with TACE and RFA. They found that sorafenib combined with TACE-RFA (S-TACE-RFA) was well-tolerated and safe. It was better than sorafenib only at improving the survival of patients with advanced HCC following primary hepatectomy. Zhu et al.^111^ conducted a retrospective analysis of the clinical data generated following the administration of this therapy to patients with medium and large hepatocellular carcinoma. The results showed that patients receiving S-TACE-RFA had a longer median RFS than those receiving TACE-RFA (24.0 months vs 10.0 months, P ​= ​0.04) and a better median OS (63.0 months vs 36.0 months, P ​= ​0.048). In addition, Chen et al.^104^ reviewed the status quo of RFA combined with multimodal therapies for the treatment of HCC.

In July 2020, Huang et al.^112^ reported a case of targeted therapy plus immunotherapy combined with multi-intervention for treatment of advanced HCC. The patient was a 55-year-old man with a history of HBV and Child-Pugh A liver function and retroperitoneal lymph node metastasis at the first consultation. From June 17, 2017 to July 21, 2019, based on the patient's changing medical condition, they successively conducted TACE of primary lesion, TACE of intrahepatic metastasis, oral treatment with sorafenib toluene sulfonic acid tablet, microwave ablation, I125 implantation for cervical lymph node metastasis lesion, intravenous drip of nivolumab, oral administration of regorafenib, and a second TACE treatment of lesions. The patient was followed up until November 1, 2019, and his condition was stable, with a survival time of more than 2 years. The patient showed resistance to sorafenib and positive PDL1 gene expression. The medical team adjusted and increased the treatment regimen accordingly based on these changes; hence, this can be regarded as a model for individualized HCC treatment.

## 7 Summary

Locoregional therapy, immunotherapy, and targeted therapy have their own merits in the clinical treatment of hepatocellular carcinoma, but their individual efficacies are not satisfactory. However, combining two or more approaches has shown promising results, especially at improving the effectiveness and survival times for patients with HCC, and can even have an impact on classic first- and second-line therapies, such as surgery, TACE, and sorafenib. Therefore, the use of combination therapy in HCC treatment requires further research. The literature review also showed that future research is required. We hypothesized that TACE could be carried out using nanomaterial-based DEB, combined with multiple targeted drugs, anti-tumor drugs, and immune or metabolic checkpoint inhibitors. With a combination of multiple approaches, TACE is expected to effectively kill HCC lesions. Under the premise of controlling side effects, the quality of life of patients is greatly improved, and the life of patients is prolonged. This is not an all-or-nothing “cocktail” but rather a quest for a standard treatment such as TB-combination chemotherapy. In future, the clinical treatment strategy for HCC will definitely change from a simple conventional treatment to combined and comprehensive treatments, and will eventually evolve into individualized treatment based on the patients' own conditions, providing hope for patients with HCC.

## Declaration of competing interest

We declare that we have no financial and personal relationships with other people or organizations that can inappropriately influence our work.
